# An attention-augmented lightweight convolutional framework for fine-grained plant leaf disease classification

**DOI:** 10.3389/fpls.2026.1762956

**Published:** 2026-02-09

**Authors:** Adithiyaa D, Lakshhmi Narayanan T, Manas Ranjan Prusty

**Affiliations:** 1 School of Computer Science and Engineering, Vellore Institute of Technology, Chennai, India; 2 Centre for Cyber Physical Systems, Vellore Institute of Technology, Chennai, India

**Keywords:** fire module, hybrid block, lightweight CNN architecture, spatial attention block, squeeze-and-excitation block

## Abstract

In the recent era, the growth of deep learning is inevitable. Various models such as convolutional neural networks (CNNs) and transformers are used widely in images for high classification accuracy. Since the invention of transformers, researchers have widely used novel approaches using transformers to achieve an impressive accuracy. In spite of this, this paper proposes a novel custom lightweight CNN model called Attentive and Lightweight Network (ALNet). ALNet consists of three major blocks: stem, core, and head. The core part is the novel classifier built as an inspiration from various pre-trained models such as ResNet, SENet (Squeeze and Excitation Network), EfficientNet, SqueezeNet, and ShuffleNet. The main objective is to build a model that has a high classification accuracy while reducing the number of parameters. This reduces the size of the model and hence makes it easy to deploy on cloud platforms and use in edge devices. The model was evaluated using 5-fold cross-validation on three different datasets. The primary dataset was a grapevine dataset with an accuracy of 99.78 percent and 100 percent in multi-class and binary classification respectively. To test the robustness of the model, a multi-class classification using the apple dataset achieved an accuracy of 99.95 percent and a binary classification with the cherry dataset achieved an accuracy of 100 percent. ALNet uses only 0.17 million parameters which is 18 times less parameters than the lightest model (SqueezeNet) and it takes only 14 seconds to train each epoch while pretrained models take 17–31 seconds. ALNet requires only 151.98 MFLOPs with a model size of 677.20 KB, making it approximately 18 times smaller than SqueezeNet. On the whole, ALNet is a highly accurate, lightweight model for plant leaf diseases prediction.

## 1 Introduction

Global food security is under constant pressure from agricultural pathogens, which cause plant diseases that reduce both yield and quality of crops. According to the Food and Agriculture Organization (FAO), plant pathogens are responsible for losses of nearly 20–40 percent of global crop production annually, amounting to an estimated economic loss of over USD 220 billion per year (John et al., 2023). These threats are even more severe in developing countries, where these infectious diseases in crops have resulted in catastrophic yield reductions, with some causing 80–90 percent losses in staple crops. Such events severely impact local economies and make hunger and poverty situations worse in rural areas, where agriculture is the primary livelihood source (Vurro et al., 2010). Effective disease control is further complicated by the need for integrated approaches that combine epidemiology, agronomy, and agricultural practices (e.g., organic methods, fertilizers) to mitigate pathogen spread and crop damage across diverse agroecological zones (Khoury and Makkouk, 2010).

Traditional methods of disease identification are majorly based on visual inspection by experts, which is labor-intensive, time-consuming, and highly prone to error, especially when dealing with early-stage symptoms or slight visual variations. The emergence of the latest technologies like deep learning has revolutionized image classification tasks like disease identification in plants. For instance, Mohanty et al. (2016) demonstrated that convolutional neural networks (CNN) trained on the PlantVillage dataset achieved classification accuracies exceeding 99 percent, setting a benchmark for agricultural image analysis. Subsequent works have extended this approach to field images with more complex backgrounds, even though the accuracy often decreases in uncontrolled environments (Ferentinos, 2018).

However, the pursuit of maximum accuracy of deep learning models presents a significant real-world constraint. Architectures renowned for top performance, such as Visual Geometry Group (VGG) and ResNet, frequently contain tens or even hundreds of millions of parameters, which require large storage space in memory and high computational power for real-time deployment, and hence are usually run on PCs with high-end GPUs. This dependency contradicts the requirement for a practical, diagnostic tool capable of functioning directly on resource-constrained edge devices, such as low-power microcontrollers or standard mobile phones, in rural environments where cloud connectivity is often unreliable.

Lightweight neural networks are CNNs with fewer parameters which reduce the memory and training time. Hence, they have gained attention for their notable functionality of being compact enough to be able to deploy in resource-constrained devices, such as mobile phones and embedded devices. Models such as ShuffleNet, SqueezeNet, and EfficientNet provide parameter-efficient architectures with competitive accuracy (Khan et al., 2023). However, these networks are generally designed for generic object recognition and may not exactly identify the fine-grained disease patterns in agricultural datasets. Recent research has also discovered that the use of attention mechanisms (Duhan et al., 2024), such as channel and spatial attention, has been employed to enhance feature selection in leaf disease classification tasks.

Despite these advances, there remains a significant gap in terms of accuracy between the model being highly accurate but computationally heavy (ResNet, VGG) in comparison with lightweight models optimized for real-world deployment. This trade-off between accuracy and efficiency forms the central challenge in developing practical plant disease classification systems. Addressing this gap is significant not only for creating high-performance AI-based systems but also for enabling the easy incorporation in day-to-day lives of farmers worldwide using low-edge devices.

This paper presents the ALNet model, a custom lightweight CNN architecture specifically designed to bridge this gap for leaf disease classification. ALNet’s contributions:

- Novel Lightweight CNN Architecture: Proposes ALNet which combines various pretrained model blocks for an enhanced feature learning with minimal parameters.
- High Parametric Efficiency: Achieves only 0.17M parameters and 151.98 MFLOPs, enabling lightweight deployment on resource-constrained devices.
- Compactness: Model size of 677.20 KB demonstrating exceptional model compactness.
- Proven Generalizability: Effectively validated on grape, apple, and cherry leaf disease datasets, confirming robustness and adaptability to diverse agricultural dataset.

The organization of the remaining paper is structured as follows: Section 2 presents related work in other related deep learning models, lightweight models and attention mechanisms. Section 3 specifically highlights the novelty of our work along with the motivation. Section 4 details the complete architecture of the proposed ALNet model. Section 5 describes the datasets used for validation. Section 6 explains the experimental setup and the evaluation metrics used. Section 7 presents and analyzes the comprehensive results, comparing ALNet against baseline models, across all three datasets. Finally Section 8 concludes the paper, summarizing our contributions and discussing potential directions for future research.

## 2 Literature review

For classifying various plant leaf diseases, various Machine Learning (ML) models, Deep Learning (DL) models, and hybrid models were discussed in a review paper (Ngugi et al., 2024). Traditional ML models such as Random Forest (RF), K-Nearest Neighbour (KNN), and Support Vector Machine (SVM) were used along with feature selection techniques such as Principal Component Analysis (PCA), Gray-Level Co-occurrence Matrix (GLCM), and color histograms. Standard custom CNNs are adopted and achieve 95–99 percent accuracy. Transfer learning is used to train pre-trained models such as VGG, ResNet, and so on. For real-time disease spotting, various object detection techniques such as You Only Live Once (YOLO) and Faster R-CNN are used. Generative Adversarial Networks (GANs) are used for image augmentation in various papers, while autoencoders are used for anomaly detection and denoising. For improving the feature extraction, transformers, such as vision transformers or Swin transformers, are used. Capsule networks are used for hierarchical features and viewpoint invariance. Various ensemble models are custom built by coming two or more models together for increasing the robustness of the model.

Harakannanavar et al. have compared the accuracy of different models such as SVM, KNN and CNN for classifying the tomato leaf diseases (Harakannanavar et al., 2022). They have followed a three stage pipeline where they have preprocessed the images in the first step by resizing and histogram equalization. Then, data was segmented using K-means clustering and contour tracing. Finally, the features were selected using discrete wavelet transform, PCA and GLCM. These data were then sent to SVM, KNN and CNN for classification and achieved an accuracy of 88, 97 and 99.6 percent respectively. Sharma et al. highlights the issue with CNNs in predicting poorly on independent or unseen datasets (Sharma et al., 2020). This paper compares two methods of F-CNN and S-CNN where F-CNN trains the model on full leaf images while the S-CNN trains the model on segmented leaf images. The research has shown that the CNN predicts much better when the image is segmented. The accuracy achieved in S-CNN is 98.6 percent. They have trained on the tomato leaf diseases from the PlantVillage dataset. A semi-automated system for grapevine leaf diseases classification emphasis on the pipeline of image segmentation, hybrid feature extraction and ensemble classification (Kaur and Devendran, 2024). Grey wolf optimization was used as an optimization of K-means clustering. Features were extracted using law’s mask, grey level co-occurrence matrix, low binary pattern and gabor features. They have used various ensemble models to classify the images and achieved a highest accuracy of 95.69 percent. UnitedModel (Ji et al., 2020) is a combination of GoogLeNet and ResNet50 which uses transfer learning to train on the grapevine dataset. Augmented dataset was used to create the feature map from both these models. This was then sent to the global average pooling, concatenation, dropout and fully connected layer and softmax output. This achieved an accuracy of 98.57 percent.

A comparative study was carried out on 14 CNN architectures and 17 vision transformers on the grapevine leaf disease dataset and grape variety classification (Kunduracioglu and Pacal, 2024). The study was carried out to check whether vision transformers gave higher accuracy or CNNs gave higher accuracy. Two models which include Swin transformer and Inception-V4 achieved an accuracy of 100 percent. It was concluded that CNNs were performing better in small datasets and ViT had better scalability. Talaat et al. (2025) have proposed a novel plant disease detection algorithm (PDDA) for predicting diseases from grapevine leaves. The PlantVillage dataset was used in this study. The images were preprocessed by applying noise reduction, normalization and enhancement. The features were carefully selected from the preprocessed images and those were fed to the Optimised CNN to make predictions. The hyperparameters were turned based on the fuzzy logic and grid search. The accuracy achieved is 99.7 percent. Shantkumari and Uma (2023) proposed to use two classifiers to predict grapevine leaf diseases. The images were preprocessed to remove noise and to segment images. This paper used histogram gradient features and extended histogram gradient features to train the model. CNN was employed for deep learning based prediction and improvised KNN was used to handle limited available data and reduce the complexity of the architecture. CCN and improvised KNN have accuracy of 96.60 percent and 98.07 percent respectively. The DLVTNet (Dense Lightweight Vision Transformer Network) was another unique approach to predict plant leaf disease datasets (Zhang et al., 2025). This model includes a three stage pipeline where generative models are used to augment the dataset and this dataset is LVT block for multi-scale feature fusion and was finally sent to DLVT for enhanced disease area focus. This achieved an accuracy of 98.48 percent on grape dataset and 96.12 percent on tomato dataset. This model has reduced the number of parameters by 42.7 percent of that of the MobileNetV4 while maintaining a high accuracy.

Several studies have focused on apple disease classification using deep learning. For instance, Liu et al. (2018) proposed a novel deep learning model which is a modified AlexNet that incorporates GoogleNet’s Inception module and replaces fully-connected layers, achieving 97.62% accuracy. They address the challenge of limited data by generating a large dataset of 13,689 images using techniques like rotation, brightness adjustment and PCA jittering, and they demonstrated that this technique of data generation improved their model’s accuracy by 10.83% with 51.2 million fewer parameters than AlexNet. Bansal et al. (2021) proposed an ensemble approach using deep learning by combining three pre-trained models (DenseNet121, EfficientNetB7, and EfficientNet NoisyStudent) via model averaging. They trained their model on the Plant Pathology 2020 Dataset which included a difficult ‘multiple diseases’ category, and their model achieved an overall accuracy of 96.25%, correctly identifying 90% of cases of ‘multiple diseases’ class, compared to only 54-69% accuracy achieved by the individual models, emphasizing the benefit of ensembling for better classification.

To address the need for efficient models deployable on mobile devices, Gao et al. (2024) proposed a lightweight apple leaf disease detection model named YOLOv8n-GGi, based on YOLOv8n. They trained their model on the AppleLeaf9 dataset expanded by data augmentation and their model achieved 86.9% mAP(mean average precision) and was 3.4% more accurate in detecting the disease compared to YOLOv8n. The key modifications in their model includes replacing standard convolutions with GhostConv and C3Ghost modules for making the model lightweight, which significantly reduced the model size to 3.8MB and the model also integrates a Global Attention Mechanism(GAM) and an improved BiFPN for better feature fusion and accuracy. Fu et al. (2022) proposed a novel, lightweight CNN that systematically modifies AlexNet architecture to create their lightweight model which is 37 times smaller than AlexNet with 5.87MB model size and a validation accuracy of 97.36%. They trained their model on publicly available AI Studio data that contains 26,377 images of apple leaves with simple and complex backgrounds. For their model, they re-engineered the original AlexNet architecture to incorporate Global Pooling Layers, Dilated Convolution and Attention(SE) modules. Vishnoi et al. (2023) proposed a lightweight CNN model named Conv-3 DCNN for detecting apple leaf diseases using the PlantVillage dataset to identify Apple Scab, Black Rot, and Cedar Rust. The Conv-3 DCNN consists of only three convolutional layers(with 32, 16, and 8 filters respectively), each followed by max-pooling. Their model achieved 98% accuracy with only ~1.2 million parameters and an 11MB size, demonstrating the feasibility of an accurate lightweight model for deployment.

A custom deep CNN which uses some standard data preprocessing techniques and hyperparameter optimization techniques (Jiang et al., 2025). This model uses the cherry dataset and it takes only 72s to train one epoch. The overall accuracy of the model is 99.2 percent. A hybrid method to improve the classification of cherry leaf diseases is by using CNN for feature selection and gradient boosting for classification (Sharma and Minhas, 2024). The dataset was augmented and normalized and then the disease severity level of each spot was labelled manually. Hence this model was trained to predict the severity of the cherry leaf disease with an accuracy of 98.9 percent. Precision, recall and F1 score were also high indicating a balanced performance of the model on all the classes. Another research paper has given a novel architecture for cherry leaf disease segmentation and feature extraction (Gupta et al., 2017). The images were fed to preprocess by removing different channels and then threshold and morphological operators were used to remove noise and other irrelevant details. The powdery mildew disease regions were correctly identified using CLAHE.

A lightweight mobile friendly model was proposed by Tang et al. (2020) where the backbone is shufflenet with squeeze and excitation blocks acting as channel attention mechanisms. The model accuracy on the grapevine leaf diseases dataset was 99.15 percent and it outperformed the traditional mobilenet and squeezenet. This model achieves an accuracy near to AlexNet. For a real time deployment, Faster DR-IACNN was proposed (Xie et al., 2020) which used double Risk Priority Number for stronger multiscale feature extraction. The backbone for the network was INSE-ResNet which is a combination of Inception-v1, Inception-ResNet-v2 and SE blocks. This network was tested on a custom grapevine dataset taken from lab conditions as well as from real vineyards. The mean precision was 81.1 percent. A hybrid model made from Swin transformer and GoogLeNet is SwinGNet (Nuthalapati et al., 2025). This hybrid fusion helps to achieve feature selection and feature extraction along with the hierarchical dependencies. This was deployed on Raspberry Pi 5 for real time disease detection. A custom dataset with all the existing datasets was built by the author for testing and achieved a validation accuracy of 99.2 percent. The model achieved an accuracy of 100 percent in real field tests with low latency.

By leveraging lightweight architectures such as SqueezeNet and EfficientDet-Lite0, few researchers are enabling real-time plant disease detection on low-power devices which facilitate rapid field level interventions with minimal computational overhead and achieve an accuracy of 97.89% (Akuthota et al., 2024). Models such as the Hybrid Plant Disease Classification Network (HPDC-Net) demonstrate that integrating dual-path adaptive pooling with attention mechanisms allows for processing speeds exceeding 400 FPS on GPUs, outperforming traditional deep learning frameworks in both scalability and precision. This system achieves an accuracy greater than 99% on three different datasets (Asghar et al., 2025). Utilizing exclusive model quantization to reduce memory overhead allows lightweight models to operate on low-configuration microcontrollers, providing a scalable and cost-effective model. Such CNNs achieve an F1 score of 98% indicating that such a lightweight model does not compromise performance (Rakib et al., 2024). Recent studies have introduced the PlaNet model which is a DCNN-based framework which demonstrates high-performance diagnostics. The model achieves 97.95% accuracy and 0.9752 AUC which highlights the model’s superior performance (Khanna et al., 2024). V^2^ PlantNet is a modified MobileNet-based architecture that utilizes depthwise separable convolutions and multi-stage feature extraction. By reducing the parameter count to just 389,286, this model achieves a high test accuracy of 98% while maintaining a compact size of 1.46 MB. This proves that lightweight designs can match the precision of larger, computationally expensive models (Nnamdi and Abolghasemi, 2025).

## 3 Motivation and contributions

The following section establishes the rationale for this paper. We begin by discussing the motivation to address the accuracy-efficiency trade-off and then detail the novel contributions of our proposed architecture.

### 3.1 Motivation

The increasing demand for a powerful image classification model, specifically in fields like agricultural technology, requires solutions that are both highly accurate and computationally efficient. To tackle this significant challenge, this paper introduces a custom lightweight model named ALNet (Attentive and Lightweight Network). Our design philosophy is to synthesize the most effective and efficient building blocks from established models to create a novel architecture. Our work primarily consists of two goals:

- To achieve superior classification performance comparative to all the present models across multiple datasets in PlantVillage Dataset for leaf disease classification.
- To minimize the number of trainable parameters as much as possible to enable smoother and faster deployment on any resource-constrained devices such as mobiles, edge devices, and also cloud deployments.

### 3.2 Novelties and contributions

ALNet offers an innovative approach to leaf disease classification, distinguished by several key contributions:

- A New CNN Architecture: This paper presents a novel CNN architecture, ALNet, offering a robust and enhanced alternative to the existing models. The primary innovation of ALNet is in its core building block, which strategically combines the parameter-efficient structure of the Fire module of SqueezeNet with the advanced Squeeze-and-Excitation and Spatial Attention mechanisms of SENet and also the residual connections of ResNet. This fusion of design principles allows the network to learn rich, meaningful features with significantly fewer parameters and hence focusing on building problem-specific and designing an efficient architecture rather than relying on large, pre-trained models.
- High Parametric Efficiency: One of the main goals was to create a model optimized for practical deployment. With a total of only around 0.17 million learnable parameters, ALNet is an extremely lightweight model as compared to other CNN models and hence can be easily deployed on resource-constrained platforms. This high-degree of efficiency is achieved through careful optimization of architecture and the appropriate integration of techniques like channel shuffling and separable convolutions, which make ALNet an impactful solution for real-world applications.
- Proven Generalizability: The performance of ALNet is validated across three different types of plant leaf disease datasets (grape, apple, and cherry), proving its adaptability and effectiveness for visual classification tasks for similar types of datasets.

## 4 Proposed architecture

ALNet, was developed based on detailed and thorough analysis of the structure of various renowned CNN models, including ResNet, SqueezeNet, SENet, EfficientNet, and ShuffleNet. By taking inspiration from these specific six models, this paper devises a novel hybrid block that serves a main part of the core module (Core Block) of the ALNet model.

**Figure 1:**
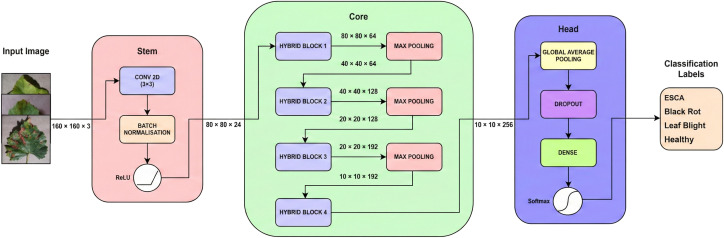
Proposed architecture diagram of ALNet.

As illustrated in **Figure 1**, the final architecture consists of three main parts:

- an initial feature extraction module (Stem Block),
- a series of core processing layers built using custom Hybrid Blocks (Core Block),
- a final classification module (Head Block).

This custom CNN architectural design allows the network to progressively learn complex features from the input images before making a final prediction. The sequential flow of the ALNet architecture and its corresponding output shapes and parameters are presented in **Table 1** below. This table clearly illustrates the summary of the entire process. As the data moves from the Stem to the Core, you can observe two noticeable changes in the output shape column. One is that the spatial size (i.e 160 x 160) gets progressively smaller. And the second one is that the channel depth (the last number, like 24, 64, 128) gets progressively larger. This implies that the network will learn simple features first, and then combine them into complex features (like leaf shapes or disease spots). Finally, the output shape of the Head block, (None, 4) represents the model’s final output. The None here represents a flexible placeholder for the batch size (the number of images processed at once) and the 4 in the output shape corresponds to the number of classes in the dataset. For each image, the model outputs four numbers, representing the final probabilities for each class. The detailed description of how each block is structured is explained in the following sections.

**Table 1:** Transition list of proposed ALNet.

Stages	Block Type	Layers	Output shape	Parameters
Input	Input	Input	160 x 160 x 3	0
Stem	Feature Extraction	Conv2D + BN + ReLu	80 x 80 x 24	744
Core	Hybrid Block 1	Fire + SE + Spatial Attention + Residual Path	80 x 80 x 64	4610
	Max Pooling	MaxPooling2D	40 x 40 x 64	0
	Hybrid Block 2	Fire + SE + Spatial Attention + Residual Path	40 x 40 x 128	19618
	Max Pooling	MaxPooling2D	20 x 20 x 128	0
	Hybrid Block 3	Fire + SE + Spatial Attention + Residual Path	20 x 20 x 192	50882
	Max Pooling	MaxPooling2D	10 x 10 x 192	0
	Hybrid Block 4	Fire + SE + Spatial Attention + Residual Path	10 x 10 x 256	96482
Head	Classifier	Global Avg. Pool + Dropout + Dense	(None, 4)	1028
**Total Parameters**				**173364**

Bold values denote major findings in the work.

### 4.1 Stem block

The Stem Block serves as the initial feature extraction module, designed for initial processing of the raw input image. As shown in **Figure 1**, it consists of a sequential stack of three layers which are a convolution layer, a Batch Normalization Layer, and a ReLU (Rectified Linear Unit) activation function. First, a 3 x 3 convolution with a stride of 2, processes the raw input image which serves the dual purpose of performing early feature extraction and finds basic patterns like edges or colors and also it downsizes the image size from 160 x 160 to 80 x 80 pixels.

After this, the Batch Normalization Layer, which is the second layer, stabilizes the learning process by normalizing the outputs of the convolution layer. This re-centers and re-scales the feature maps which helps to mitigate the problem of internal covariate shift, allowing the model to train faster. Finally the ReLU activation function is used. This simple yet powerful function, defined as f(x)=max(0, x) introduces non-linearity into the network enabling the model to learn complex and abstract patterns in the data. This also helps to address the issue of vanishing gradient problem making it a standard choice for deep networks.

### 4.2 Core block

The Core Block forms the main body of the ALNet architecture and comprises a series of four repeated, custom-designed Hybrid Blocks. The selection of exactly four blocks was a decision made after multiple experimentations to balance the need for sufficient network depth for the model to learn intricate disease patterns, along with the objective of maintaining a lightweight architecture. Using fewer hybrid blocks might lead to underfitting of the model and poor pattern detection, while adding more hybrid blocks is not advantageous as well, since, although the model will learn more complex patterns by increasing the count of hybrid blocks, it might lead to overfitting of the model, along with a substantial increase in the number of parameters of the model and computational cost. The final output of the Core Block is an aggregated feature representation that serves as the input to the classifier head.

#### 4.2.1 Hybrid block

**Figure 2:**
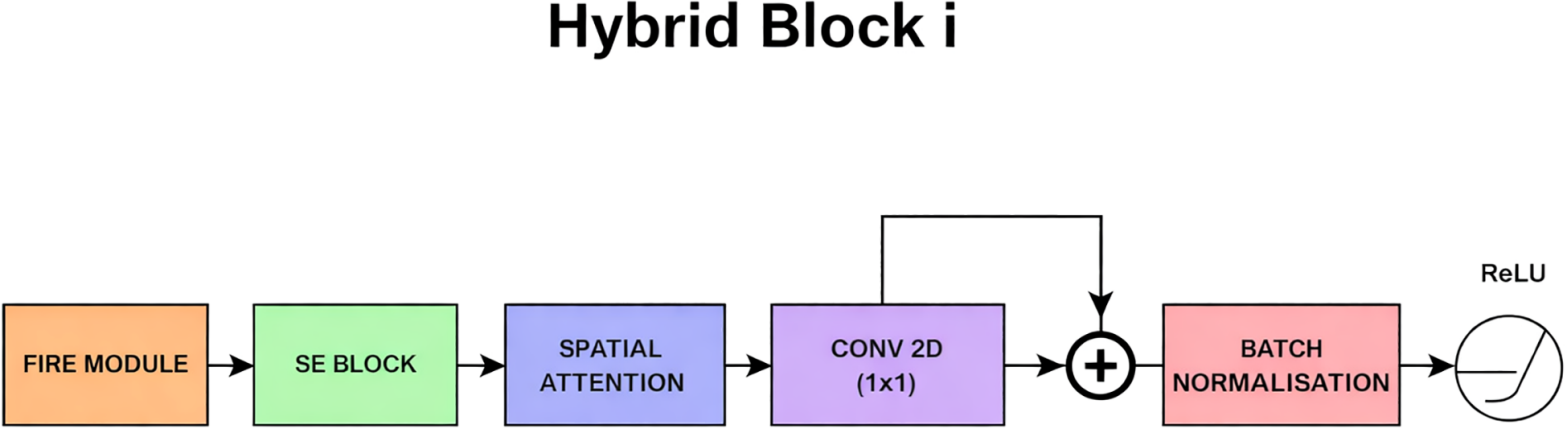
Internal blocks in each hybrid block.

The Hybrid Block is the primary innovation of ALNet, which strategically combines parameter-efficient, attention-based principles. Its internal structure is detailed in **Figure 2**. The purpose of the Hybrid Block is to facilitate the streamlined training of a neural network by using a residual connection, a core principle of ResNet. This mechanism allows the model to learn features based on the residual function rather than a simple, raw feature mapping. The output of a hybrid block can be expressed as shown in **Equation 1**:

(1) Y = σ(F(X) + G(X))

Where X is the input feature map, F(X) represents the output of the main path(Fire Module, SE Block and Spatial Attention), and G(X) is the residual path, The function σ denotes the ReLU activation.

In the model design, G(X) is a direct identity mapping for the residual path, and it does not transform the data in any way at all and simply takes the original input from the beginning, resizes it using a 1 x 1 convolution, to ensure that the original information is preserved, even if the main path’s output is not perfect. The main path is where the network does all the heavy processing through a sequence of Fire Module, SE Block, and Spatial Attention. It takes the input data and transforms it completely, extracting more abstract features.

##### 4.2.1.1 Fire module

**Figure 3:**
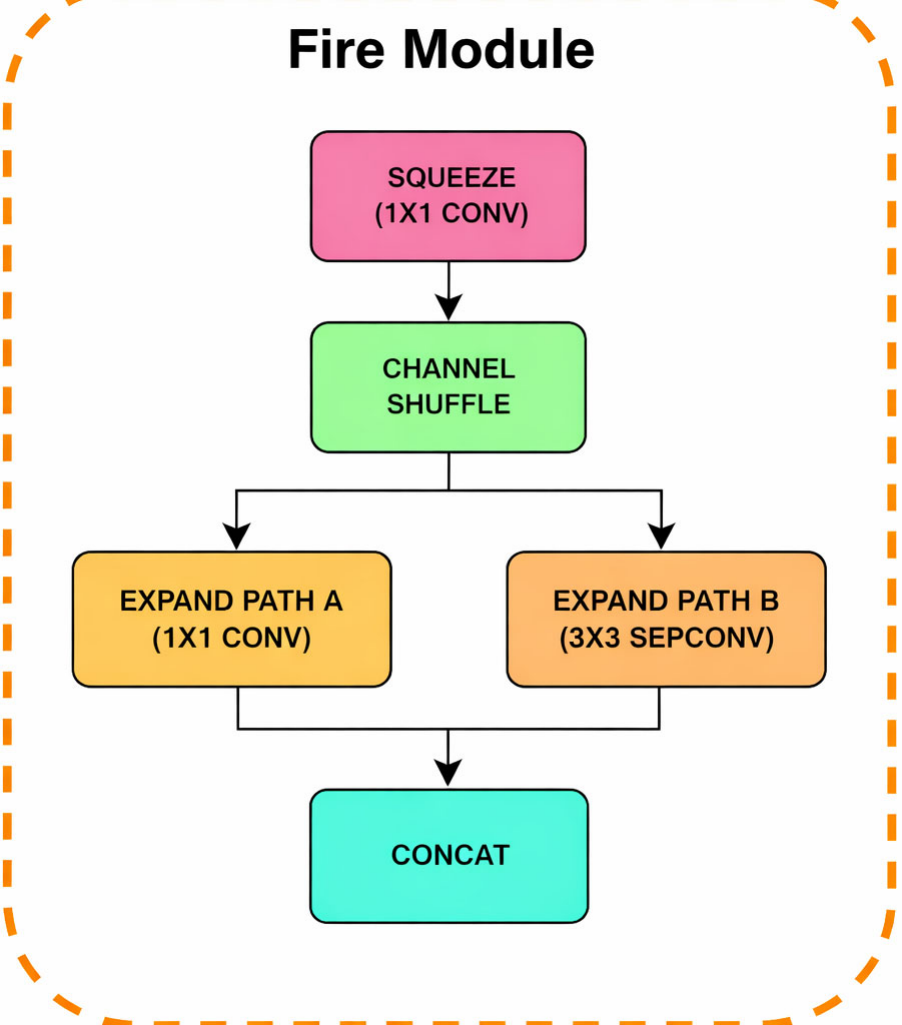
Internal layers in fire module.

The fundamental unit within the Hybrid Block is the custom Fire Module, shown in detail in **Figure 3**. This module is inspired from Squeezenet’s original design by incorporating key improvements. Initially when the data is sent through the fire module, it is processed through a 1x1 convolution(Squeeze) which reduces the channel dimensions. This is immediately followed by a Channel Shuffle operation (inspired by ShuffleNet), which improves feature selection among groups of channels. The output is then split into two parallel paths: a 1 x 1 convolution (Expand Path A) and a 3 x 3 depthwise separable convolution (Expand Path B), a technique inspired by MobileNet. The results of both paths are concatenated at the end to form the module’s output.

To enable the neural network to focus on the most informative features, the Hybrid Block employs both channel and spatial attention using SE Block(Squeeze-and-Excitation block) and also the Spatial Attention Block.

##### 4.2.1.2 Squeeze-and-excitation block

This operation begins with a Global Average Pooling layer which takes the spatial dimensions of the feature map from the previous module and converts it into a single vector of numbers which is the Squeeze operation. Each number in the vector represents the average value of a single channel, providing the network with an understanding of the overall content of the feature map. The Squeeze operation aggregates global spatial information from the input feature map $u_{c}\;$(with spatial dimensions H and W) into a channel descriptor z ∈ R1×1×C using Global Average Pooling as shown in **Equation 2**:

(2) $$z_{c}=\;\frac{1}{H\;X\;W}\;\;\sum_{i=1}^{H}\sum_{j=1}^{W}u_{c}\left(i,j\right)$$

where $z_{c}$ is the output (the squeezed value) for the c-th channel, $u_{c}$ is the input feature map for that channel, and H and W are the height and width of the feature map.

**Figure 4:**
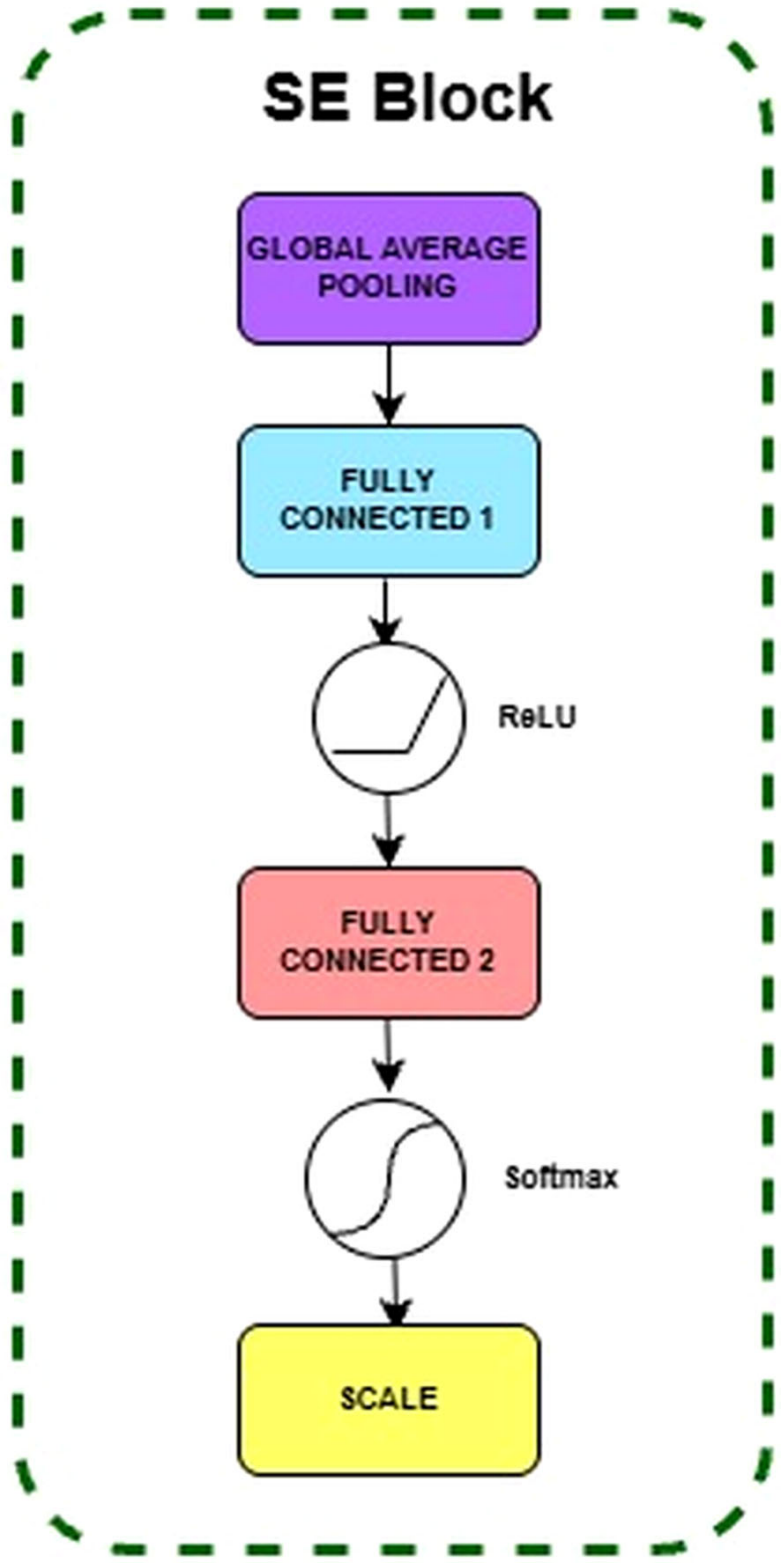
Internal layers in SE block.

The squeezed vector z is then passed through two fully connected (Dense) layers which is the Excitation operation. This is a crucial step where the network learns the relationship between different channels. After that, a Softmax activation function(Sigmoid activation) is applied on the vector containing the channel weight to convert the value to between 0 and 1. The Excitation operation is represented shown in **Equation 3**:

(3) $$s=\;\sigma \left(Dense_{2}\left(ReLU\left(Dense_{1}\left(z\right)\right)\right)\right)$$

where s is the final vector of channel scaling factors (the weights), σ denotes the Sigmoid activation function, $Dense_{1}$ and $Dense_{2}$ represent the two fully connected layers, ReLU is the Rectified Linear Unit activation, and z is the squeezed channel descriptor from **Equation 2**. Finally, the feature map is rescaled by multiplying the original output by these weights, as illustrated in **Figure 4**.

##### 4.2.1.3 Spatial attention block

**Figure 5:**
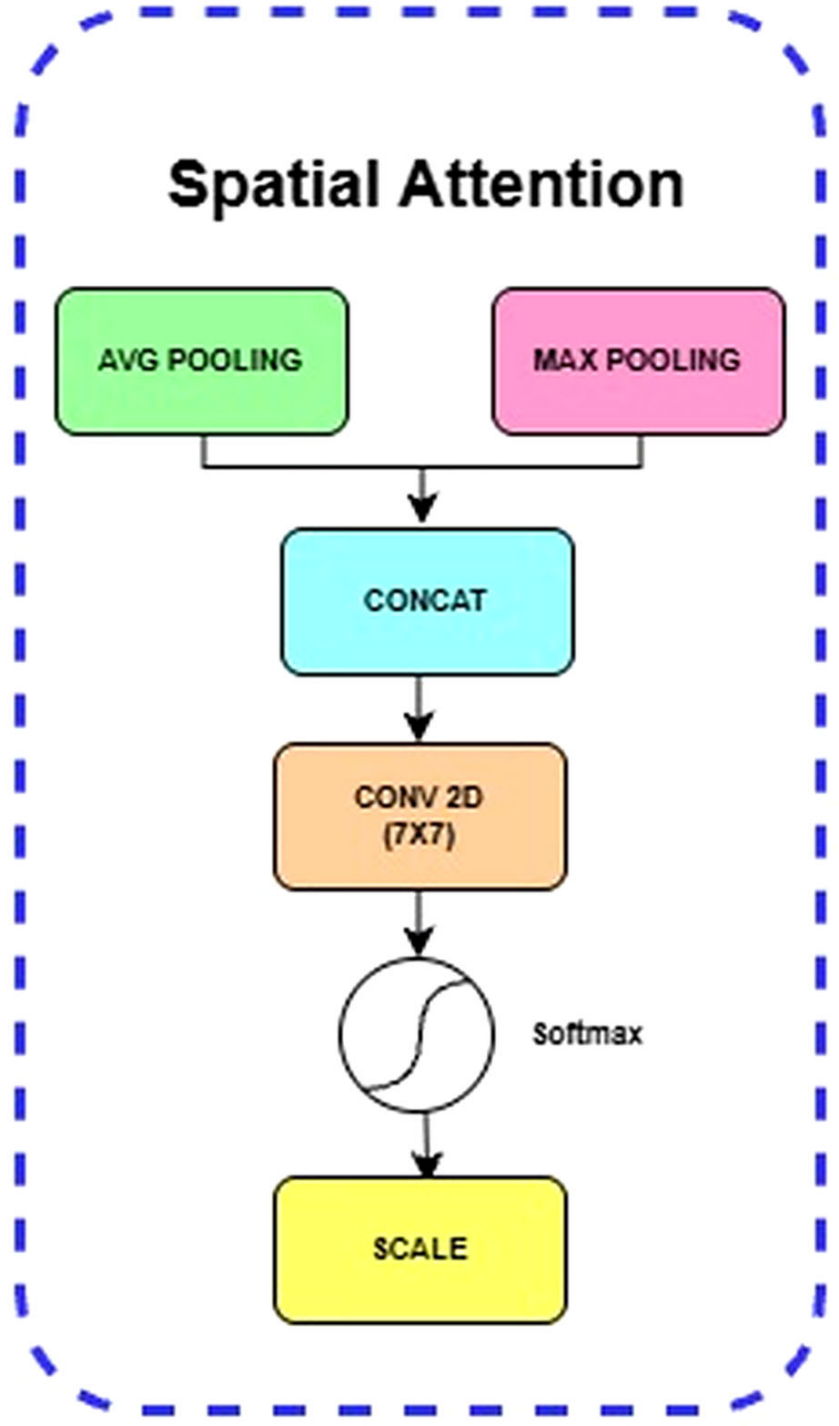
Internal layers in spatial attention block.

While the SE Block focuses on “what” is important, the Spatial Attention Block complements it by focusing on “where” the most informative features are located within the feature map. It computes average-pooled (Favg) and max-pooled(Fmax) features along the channel axis. These are concatenated and convolved to generate a spatial attention map Ms(F), as shown in **Equation 4** and depicted in **Figure 5**.

(4) $$M_{s}\left(F\right)=\;\sigma \left(Conv^{7x7}\left(\left[AvgPool\left(F\right)\;;\;MaxPool\left(F\right)\right]\right)\right)$$

where $M_{s}\left(F\right)$ is the final spatial attention map, σ denotes the Sigmoid Activation function, Conv**^7x7^** represents a single convolutional operation with a large 7 x 7 kernel, which is effective at capturing a wide spatial context, AvgPool(F) and MaxPool(F) are the feature maps generated by performing average pooling and max pooling operations, respectively, across the channel dimension of the input feature map F, […; …] denotes the concatenation operation, which combines the average-pooled and max-pooled feature maps along the channel axis into a two-channel input for the convolution layer. The final output is obtained by element-wise multiplication of the original feature map F with the attention map M_s_(F).

##### 4.2.1.4 Conv2D layer (residual path projection)

As depicted in **Figure 2**, a Conv2D layer with a 1 x 1 kernel is integrated into the residual path G(X), specifically placed just before the element-wise addition with the main path F(X). The main function of this layer is dimensionality matching and hence it is not always active. The 1 x 1 convolution acts as an efficient linear project to increase the channel depth of the input X so that its shape aligns perfectly with F(X), enabling the element-wise addition required by the residual connection.

##### 4.2.1.5 Batch normalization layer

Following the element-wise addition of the main path F(X) and the residual path G(X), a Batch Normalization(BN) layer is applied, as shown at the end of the Hybrid Block in **Figure 2**. The addition operation can significantly change the statistical distribution(mean and variance) of the activations. And hence the BN layer is applied to re-normalize these combined activations. By standardizing the mean to approximately zero and the variance to one, it stabilizes the input to the final ReLU activation function and subsequently to the next layer of block. This stabilization is important for maintaining healthy gradient flow during backpropagation and preventing issues like vanishing gradients and allowing for reliable network training.

#### 4.2.2 Max pooling

A Max pooling Layer is applied between each of the first three Hybrid Blocks. This layer works by sliding a small window (typically 2 x 2) across the input feature map and outputting only the maximum value within that window for each channel. This operation serves two major purposes in our proposed model architecture.

The first one being the Dimensionality Reduction, where this layer reduces the spatial dimensions(height and width) of the feature map by half (e.g., from 80 x 80 to 40 x 40). This significantly reduces the number of parameters and computations required in subsequent layers, contributing to the model’s overall efficiency. The second major purpose is the Feature Invariance. By selecting the maximum value, Max poling layer provides a local translation invariance which means that the network becomes less sensitive to the exact position of a feature within the window and instead focuses on presence of more noticeable features. The Max Pooling Layer is intentionally omitted for the final (fourth) Hybrid Block, as the subsequent Head Block begins with a Global Average Pooling Layer that does a much more aggressive form of spatial dimensionality reduction(down to 1 x 1). If we add a Max pooling Layer after the fourth Hybrid Block, then the spatial dimensions will reduce from 10 x 10 to 5 x 5 in this case, which is basically redundant in this case, as the Head Block can directly reduce the dimensions from 10 x 10.

### 4.3 Head block

The final part of the network, the Head Block, is responsible for producing the final classification output of the model. It consists of an initial Global Average Pooling Layer, which efficiently compiles feature maps from the final Hybrid Block into a single feature vector. It is followed by a Dropout layer for regularization, which helps prevent overfitting. The final classification is then performed by a fully-connected Dense layer along with a Softmax activation function as illustrated in **Figure 1**.

To summarize, with a total of only around 0.17 million learnable parameters, ALNet is an extremely lightweight model as compared to other CNN models and hence the main goal of being able to easily deploy the model on resource-constrained platforms is achieved. This high degree of efficiency is achieved through the careful and deliberate optimization of architecture and the appropriate integration of techniques like channel shuffling and separable convolutions, which together make ALnet an impactful solution for real-world applications.

## 5 Dataset

This study uses a part of the PlantVillage dataset which is openly accessible. The primary focus is on the diseases associated with the grapevine leaves and hence an augmented dataset available on Kaggle was used. The dataset was having separate train and test images and each of them having four distinct classes namely Healthy, Black Rot, ESCA and Leaf Blight. The dataset contains a total of 9027 images with 2115 images of Healthy class, 2360 images of Black Rot class, 2400 images of ESCA class and 2152 images of Leaf Blight class.

**Figure 6:**
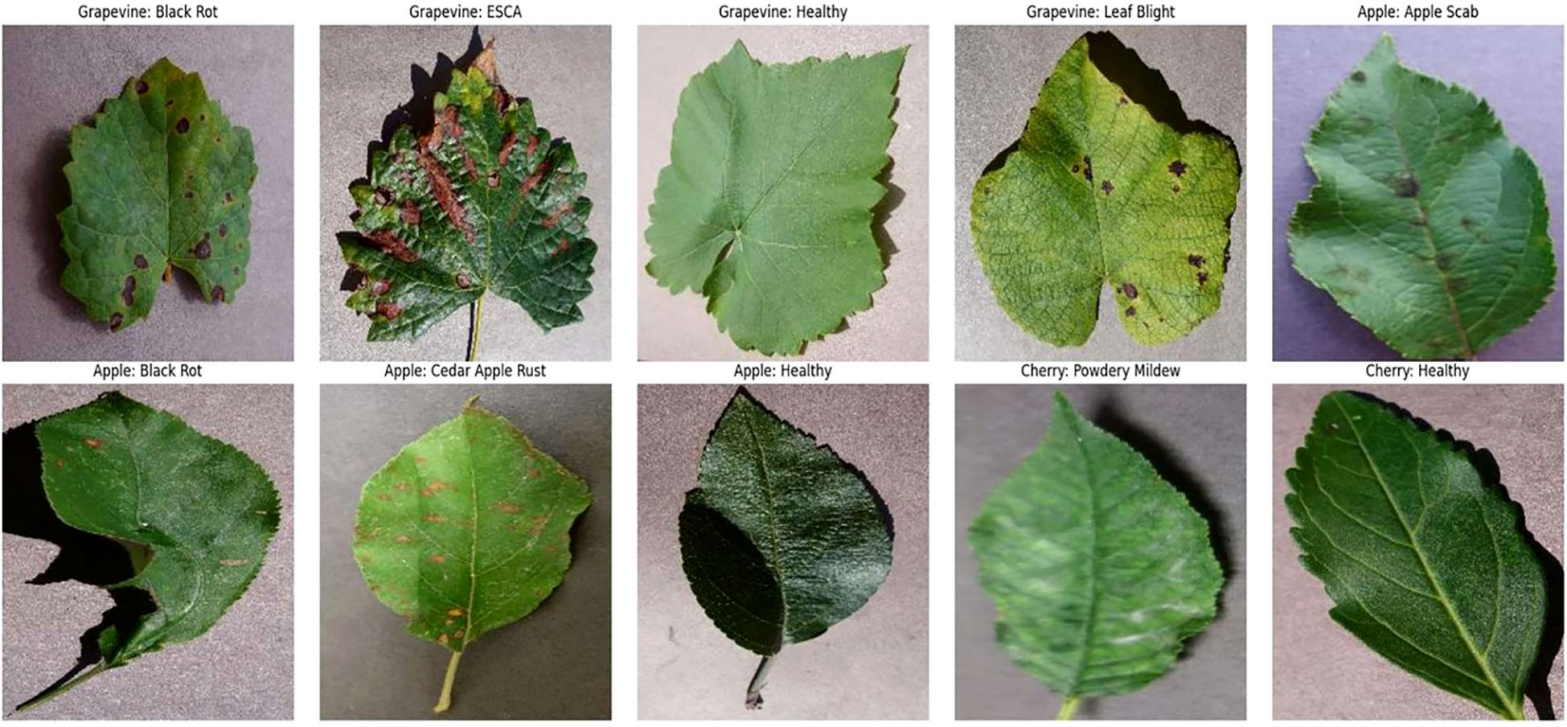
Representative samples from all the datasets.

To extend the robustness of the model, ALNet was trained and tested with two other datasets. Both these datasets are subsets from the PlantVillage dataset. One dataset is on the apple leaf diseases which had four classes of Healthy, Cedar Apple Rust, Black Rot and Apple Scab. It has a total of 9714 images with 2510, 2200, 2484 and 2520 images from Healthy, Cedar Apple Rust, Black Rot and Apple Scab respectively. Similarly, the apple dataset was taken from an augmented dataset available on Kaggle. The other one is on the cherry disease dataset which has two classes namely Healthy and Powdery Mildew. It has a total of 1906 images with 854 and 1052 images from Healthy and Powdery Mildew respectively. The cherry dataset was taken from the PlantVillage dataset and was used to carry out five-fold cross validation. Representative sample images of all the different classes in grapevine, apple and cherry datasets are shown in **Figure 6**.

**Figure 7:**
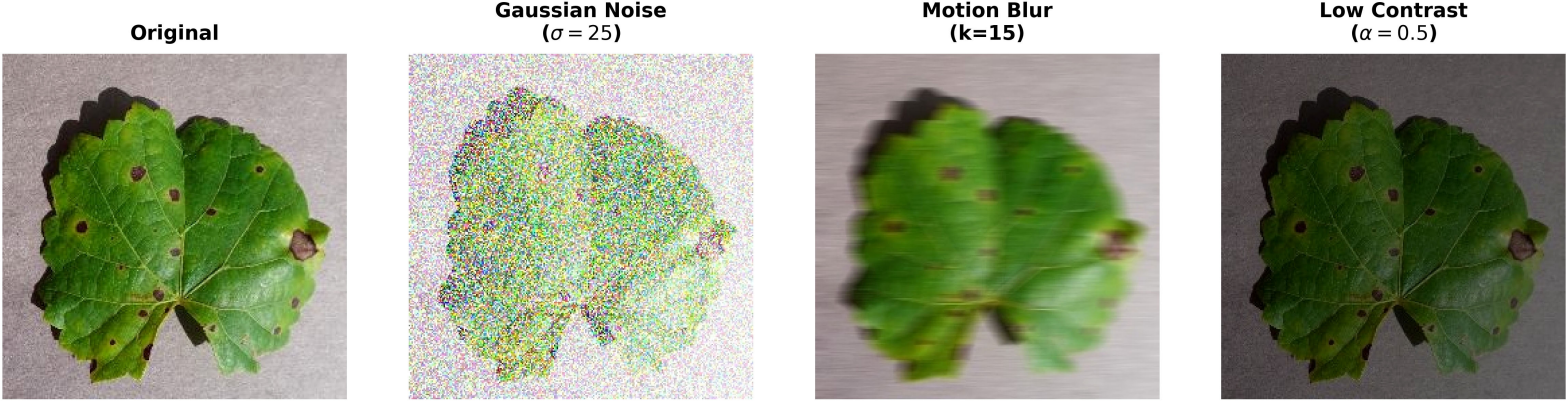
Representative samples from the grapevine noisy dataset.

Finally, to address the challenges of real-world agricultural environments that have unpredictable lighting and camera conditions and to do a rigorous evaluation of the model’s robustness, a synthetic expansion of the grapevine dataset was conducted. This Grapevine Noisy Dataset was specifically created to replicate the environmental noise and degradation in order to bridge the gap between controlled laboratory evaluations and practical deployment. For each of the 9027 original images, three different transformations were applied, as shown in **Figure 7**.

- Gaussian Noise with a standard deviation of σ = 25 was introduced to mimic interference of sensor in low-light environments.
- Motion Blur with a kernel size of 15 was applied to simulate instability during handheld image capture.
- Low Contrast was achieved by reducing the intensity values with a scaling factor of *α* = 0.5 to represent overexposed or poor visibility conditions.

Thus, the primary dataset was expanded to a dataset namely Grapevine Noisy Dataset that contains a total of 36108 images.

## 6 Experimentation

**Figure 8:**
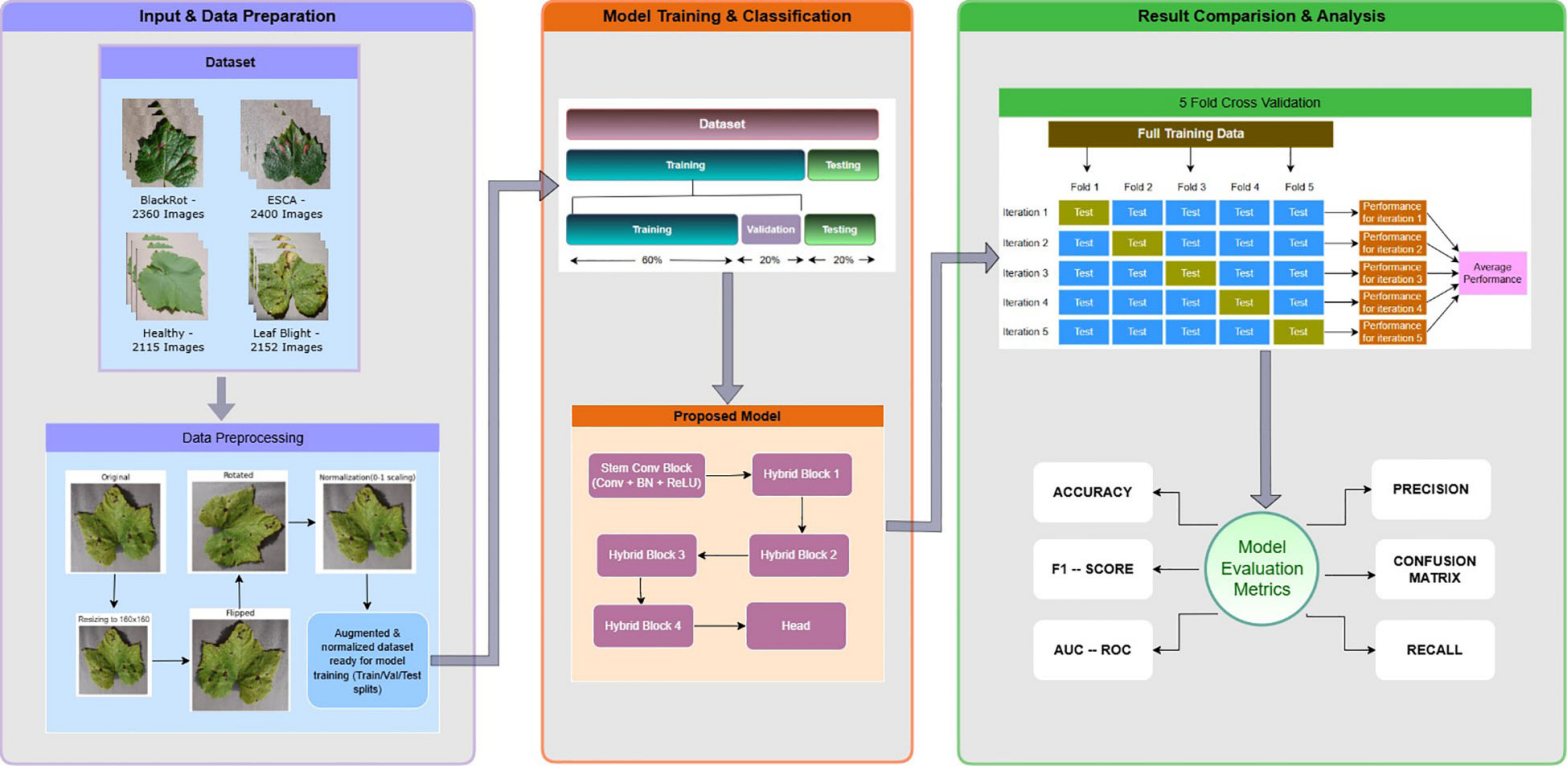
Outline of the entire training pipeline.

All the experiments were carried out on the Kaggle coding environment which uses a preconfigured Jupyter notebook with support for common machine and deep learning libraries. This paper uses GPU P100 for all the training, validation and testing to accelerate the computations and training process. This setup enabled reproducibility as Kaggle manages all the library dependencies needed for the experiments. The GPU configuration has significantly reduced the time taken for training and testing and helped us to perform multiple experiments with hyperparameter tuning in a reasonable amount of time. **Figure 8** delineates the entire training pipeline for the conducted experiment.

All models were implemented using TensorFlow/Keras. Images were resized to 160×160 pixels and normalized to [0,1]. Data augmentation including random horizontal flipping, rotation, zoom and contrast adjustment was applied during training. The model was trained using the Adam optimizer with an initial learning rate of 0.01 and sparse categorical cross-entropy loss. Training was conducted for 30 epochs with a batch size of 32. A ReduceLROnPlateau scheduler was employed to dynamically change the learning rate based on validation accuracy. For all the experiments, this paper has adopted the five-fold cross validation technique. All the datasets used were only trained using five-fold cross validation where 80 percent of data in each fold was for training and the remaining 20 percent of the data was for the validation purpose. The entire training pipeline is illustrated in **Figure 7**. The results presented in the paper include the results from the hold-out CV and 5-fold CV. The custom CNN model was trained on grapevine leaf diseases first where the performance of ALNet on a multiclass dataset was evaluated. Later, to test the robustness of ALNet on the binary classes, an equal sample of 705 from each of diseased classes (ESCA, Black Rot and Leaf Blight) created an Unhealthy class of 2115 samples and the Healthy class already had 2115 samples. To further verify the robustness of the custom CNN model, the model was trained and tested on Apple and Cherry dataset where Apple is a multiclass dataset and Cherry is a binary dataset.

In the second phase of experimentation, the model’s resilience was evaluated using the Grapevine Noisy Dataset. To maintain experimental consistency, all baseline architectures and the proposed ALNet were trained using the same five-fold cross-validation technique. The goal was to specifically test the impact of environmental artifacts like Gaussian noise, motion blur, and low contrast on the classification performance of the models. By making use of this expanded dataset of 36108 images, a comprehensive assessment of ALNet’s readiness for deployment in real-life agricultural environments is presented.

## 7 Results and discussions

In this section of the research paper, a comparative classification analysis is performed to compare the performance of the ALNet with all the common transfer learning models and state-of-the-art techniques that are currently being used in research. The evaluation is conducted across three distinct datasets: grapevine, apple, and cherry. Additionally, statistical tests are performed and the results of these are presented in this section.

### 7.1 Multi-class dataset

#### 7.1.1 Grapevine dataset

Grapevine dataset is the primary dataset on which the ALNet model was built on. **Table 2** shows the comparison of ALNet with various transfer learning models such as ResNet, EfficientNetB0, ShuffleNet, SqueezeNet and SENet on the grapevine dataset. All these models were the inspiration to create the lightweight ALNet model. The table shows ALNet is the best model with the highest mean accuracy of 0.9862, precision of 0.9868, recall of 0.9865, F1 score of 0.9863, AUC score of 0.9999. The results clearly show that ALNet outperforms all the existing transfer Learning models even though it is a lightweight model. One should also note that ALNet’s accuracy is 0.2 percent greater than the best model (SENet) and is 30.66 percent greater than the worst model (ResNet).

**Table 2:** Multi-class grapevine leaf disease prediction performance.

Models	Hold-out CV					5-fold CV				
	Accuracy	Precision	Recall	F1 Score	AUC Score	Accuracy	Precision	Recall	F1 Score	AUC Score
ALNet	**0.9978**	**0.9978**	**0.9979**	**0.9978**	**1.000**	**0.9862**	**0.9868**	**0.9865**	**0.9863**	0.9999
ResNet	0.6988	0.7263	0.6996	0.695	0.8835	0.6796	0.7067	0.6799	0.6736	0.8769
EfficientNetB0	0.9812	0.9809	0.9821	0.9813	0.9907	0.9374	0.9506	0.9386	0.9375	0.9907
ShuffleNet	0.9950	0.9949	0.9951	0.995	0.9993	0.9704	0.9731	0.9711	0.9699	0.9948
SqueezeNet	0.9823	0.9827	0.983	0.9823	0.9999	0.9527	0.9572	0.9547	0.9520	0.9998
SENet	0.9917	0.9919	0.9921	0.9919	**1.000**	0.9842	0.9848	0.9848	0.9844	**1.0000**

Bold values denote major findings in the work.

**Figure 9:**
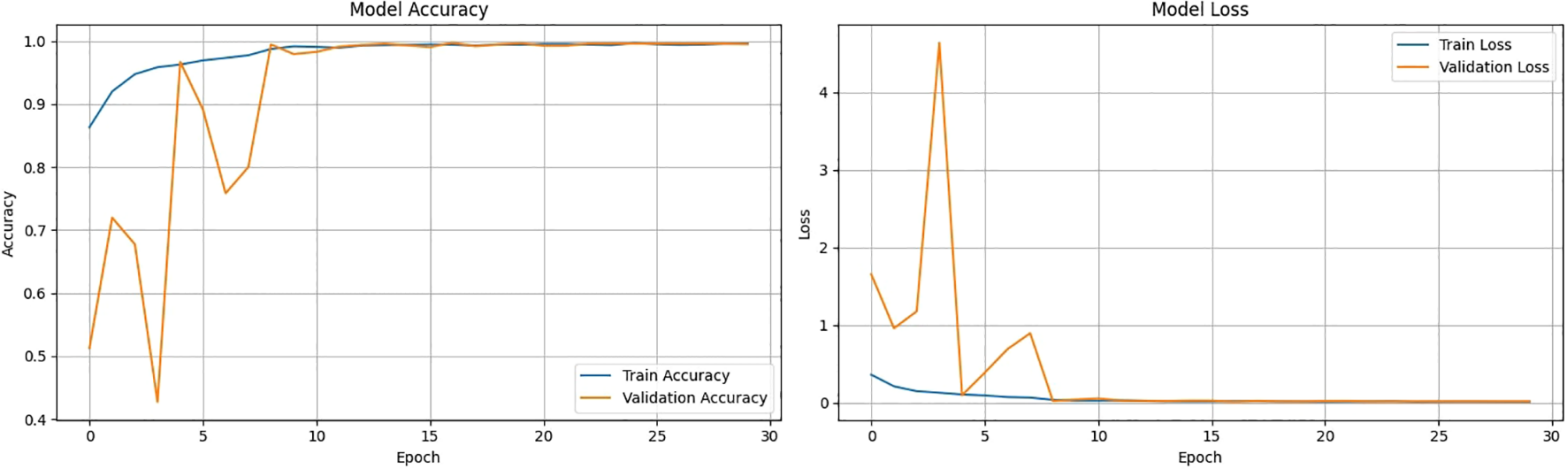
Accuracy vs. Epoch and Loss vs. Epoch graphs for ALNet.

**Figure 10:**
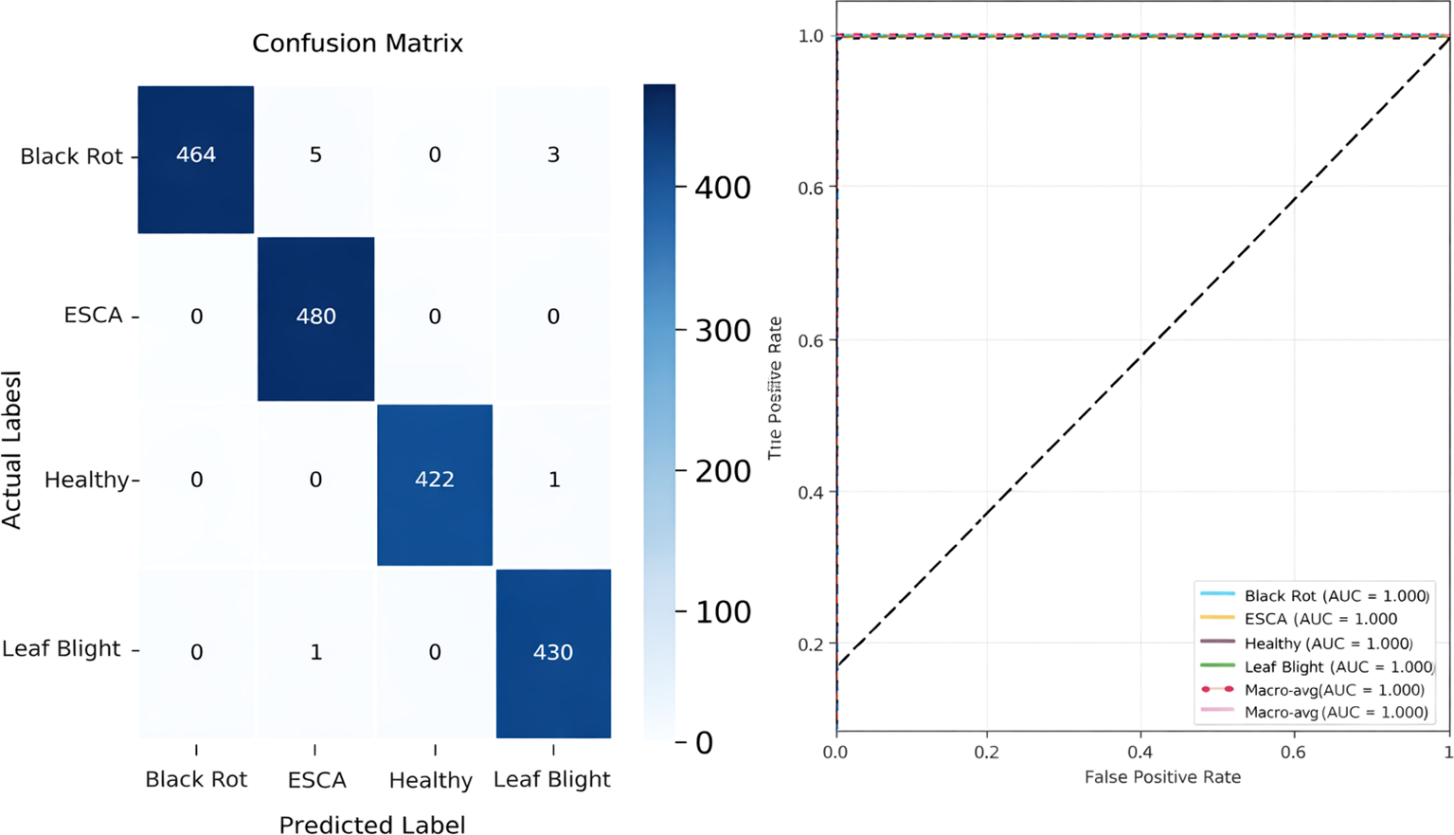
Confusion matrix and ROC curve for ALNet.

**Figure 9** shows the accuracy vs epoch and the loss vs epoch graphs. The graph clearly shows that the model is getting trained within 20–25 epochs. The training accuracy is also rising above 98 percent in 5 epochs and the training loss reduces to less than 0.2 in 5 epochs. The validation accuracy and loss are fluctuating in the initial epochs but eventually the curve gets smoother after 15 epochs and converges with the training accuracy and loss curve. **Figure 10** shows the confusion matrix for the hold out fold of ALNet. There are only 2 misclassifications in the testing data. Moreover the misclassifications are only in Black Rot and hence the precision and recall of the model for the rest of the classes are 1 indicating high reliability and efficiency in leaf disease classification. **Figure 10** also shows the ROC curve for the best fold. It is clear that the AUC value for all the classes are 1 and hence indicating that the model is robust. It is safe to claim that ALNet is 100 percent accurate being a lightweight model.

**Table 3** shows the number of parameters and the average time taken to train each epoch for various transfer learning models. ALNet uses the least number of parameters and takes the least time to train the model. The number of parameters is only 0.17 million in ALNet whereas it is in millions in all other pre-trained models. The number of parameters in the lightest model (SqueezeNet) is 18 times larger than the ALNet. Hence, ALNet is the lightest model which can predict with better accuracy. To add on, ALNet takes the least time per epoch to train the model. ALNet takes only 14 seconds while the others take between 17 and 31 seconds to train one epoch. Hence, the model can be trained faster. This implies that the model can be trained effortlessly when on edge devices. The table also shows that ALNet has the lowest model size of 677.20 KB (0.6772 MB) and requires only 151.98 MFLOPs which implies that this model is small, less computationally expensive and can be easily deployed in cloud and edge devices. The model size of ALNet is approximately 18 times smaller than the SqueezeNet which has the smallest model size among the pretrained models.

**Table 3:** Comparison of the model parameters and average time per epoch.

Models	Number of parameters (in millions)	Average Time per epoch (in seconds)	Model Size (in MB)
ALNet	**~0.17M**	**14**	**0.6772**
ResNet	~4.38M	20	90.01
EfficientNetB0	~23.59M	31	17.99
ShuffleNet	~3.60M	28	13.83
SqueezeNet	~3.11M	28	11.88
SENet	~11.28M	17	43.01

Bold values denote major findings in the work.

#### 7.1.2 Apple dataset

The apple disease dataset is a multi-class dataset with four different classes which includes the healthy, cedar apple rust, black rot and apple scab classes. **Table 4** shows the performance of various pre-trained models on apple leaf disease dataset. The table shows that ALNet is the best performing model with an accuracy of 99.78 percent, precision of 99.79 percent, recall of 99.79 percent, F1 score of 99.79 percent and AUC score of 100 percent. The least performing pre-trained model (ResNet) achieves an accuracy of 56.69 percent and the best performing pre-trained model (EfficientNetB0) achieves an accuracy of 97.56 percent. Hence, the results of ALNet on the apple dataset show that ALNet is a custom CNN which can perform well on any multi-class classification.

**Table 4:** Multi-class apple leaf disease prediction performance.

Models	Hold-out CV					5-fold CV				
	Accuracy	Precision	Recall	F1 score	AUC score	Accuracy	Precision	Recall	F1 score	AUC score
ALNet	**0.9995**	**0.9995**	**0.9995**	**0.9995**	**1.0000**	**0.9978**	**0.9979**	**0.9979**	**0.9979**	**1.0000**
ResNet	0.5847	0.5986	0.5821	0.5486	0.8350	0.5669	0.5742	0.5651	0.5347	0.8166
EfficientNetB0	0.9882	0.9888	0.9881	0.9884	0.9999	0.9756	0.9777	0.9754	0.9760	0.9997
ShuffleNet	0.9624	0.9660	0.9634	0.9636	0.9982	0.9462	0.9537	0.9478	0.9478	0.9980
SqueezeNet	0.9464	0.9499	0.9479	0.9476	0.9962	0.8775	0.8901	0.8814	0.8663	0.9787
SENet	0.9604	0.9647	0.9615	0.9615	0.9989	0.9473	0.9555	0.9490	0.9488	0.9990

Bold values denote major findings in the work.

### 7.2 Binary class dataset

#### 7.2.1 Grapevine dataset

To test ALNet on a binary dataset, the grapevine diseases dataset was used to create a binary dataset with healthy and non-healthy classes. The procedure used for creating this dataset is illustrated in the dataset section. **Table 5** shows the results for the binary classification of grapevine leaves. The results clearly show that ALNet achieves the highest performance in both the best fold and average fold. While comparing with other pre-trained models, SENet achieves the same performance as ALNet and all other models get lesser performance. The accuracy of ALNet is 11.54 percent greater than the least performing model (ResNet). The results clearly show that ALNet performs better in binary dataset too.

**Table 5:** Binary grapevine leaf disease prediction performance.

Models	Hold-out CV					5-fold CV				
	Accuracy	Precision	Recall	F1 score	AUC score	Accuracy	Precision	Recall	F1 score	AUC score
ALNet	**1.0000**	**1.0000**	**1.0000**	**1.0000**	**1.0000**	**0.9998**	**0.9998**	**0.9998**	**0.9998**	**1.0000**
ResNet	0.9019	0.9315	0.8676	0.8984	0.9653	0.8844	0.9139	0.8506	0.8798	0.9605
EfficientNetB0	**1.0000**	**1.0000**	**1.0000**	**1.0000**	**1.0000**	0.9986	1.0000	0.9972	0.9986	1.0000
ShuffleNet	0.9988	0.9976	**1.0000**	0.9988	**1.0000**	0.9811	0.9822	0.9811	0.9811	0.9997
SqueezeNet	**1.0000**	**1.0000**	**1.0000**	**1.0000**	**1.0000**	0.9995	1.0000	0.9991	0.9995	1.0000
SENet	**1.0000**	**1.0000**	**1.0000**	**1.0000**	**1.0000**	**0.9998**	**0.9998**	**0.9998**	**0.9998**	**1.0000**

Bold values denote major findings in the work.

#### 7.2.2 Cherry dataset

The cherry disease dataset is a binary dataset with two classes of powdery mildew and healthy. **Table 6** shows the performance of various pre-trained models on the cherry dataset. The results show that ALNet achieves an accuracy of 99.69 percent. While comparing with other pre-trained models, SENet achieves the highest performance with an accuracy of 99.79 percent. However, ALNet is lightweight with only 170000 parameters, the difference in accuracy is just 0.1 percent which is negligible. Hence, it is safe to claim that ALNet can perform well on any binary classification.

**Table 6:** Binary cherry leaf disease prediction performance.

Models	Hold-out CV					5-fold CV				
	Accuracy	Precision	Recall	F1 score	AUC score	Accuracy	Precision	Recall	F1 score	AUC score
ALNet	**1.0000**	**1.0000**	**1.0000**	**1.0000**	**1.0000**	0.9969	0.9970	0.9967	0.9968	0.9999
ResNet	0.9423	0.9565	0.9384	0.9474	0.9819	0.9208	0.9559	0.8982	0.9258	0.9789
EfficientNetB0	**1.0000**	**1.0000**	**1.0000**	**1.0000**	**1.0000**	0.9318	0.9953	0.8526	0.8836	0.9947
ShuffleNet	0.9948	0.9884	**1.0000**	0.9942	**1.0000**	0.9927	0.9920	0.9933	0.9926	0.9996
SqueezeNet	0.9974	0.9953	**1.0000**	0.9976	**1.0000**	0.9953	0.9962	0.9952	0.9957	**1.0000**
SENet	**1.0000**	**1.0000**	**1.0000**	**1.0000**	**1.0000**	**0.9979**	**0.9981**	**0.9977**	**0.9979**	**1.0000**

Bold values denote major findings in the work.

### 7.3 Robustness analysis on noisy and low-contrast dataset

The Grapevine Noisy Dataset which is an expanded noisy dataset augmented from the original PlantVillage grapevine leaf images is used here to evaluate the practical reliability of the ALNet model in non-ideal agricultural settings and also to assess the stability of the model when processing degraded images. **Table 7** shows the performance comparison across both hold-out and 5-fold cross-validation. Despite the increased data complexity and visual degradation, ALNet maintained a high 5-fold CV accuracy of 98.74%, outperforming SqueezeNet (97.46%) and ShuffleNet (97.21%). The least performing pre-trained model (ResNet) achieves an accuracy of 53.32%. These results demonstrate that ALNet’s custom architecture enables the network to focus on essential disease patterns even when hindered by noise or blur in the image.

**Table 7:** Performance comparison on grapevine noisy dataset.

Models	Hold-out CV					5-fold CV				
	Accuracy	Precision	Recall	F1 score	AUC score	Accuracy	Precision	Recall	F1 score	AUC score
ALNet	0.9873	0.9876	0.9877	0.9876	0.9997	0.9874	0.9879	0.9879	0.9878	0.9996
ResNet	0.5173	0.5669	0.5171	0.5027	0.809	0.5332	0.5744	0.534	0.5249	0.8093
ShuffleNet	0.959	0.9612	0.9611	0.9597	0.9987	0.9721	0.9732	0.9734	0.9724	0.9991
SqueezeNet	0.9574	0.9595	0.9578	0.9579	0.9985	0.9746	0.9753	0.9752	0.975	0.9992

### 7.4 Ablation study

To evaluate the individual contribution of each component within the Hybrid Block, an ablation study was conducted. We compared the performance of the full ALNet model against several variants where specific modules - such as the Squeeze-and-Excitation (SE) block, Spatial Attention, Residual Path, and Channel Shuffle - were systematically removed or modified. The results of this study conducted on the grapevine dataset are summarized in **Table 8**.

**Table 8:** Ablation study for ALNet.

Configuration	Parameters (M)	Accuracy	Precision	Recall	F1-score	Δ Accuracy
**Full Model (ALNet)**	**0.1734**	**99.28%**	**0.9931**	**0.993**	**0.9931**	**—**
Without Channel Shuffle	0.1734	88.43%	0.905	0.8884	0.8792	-10.85%
Without Spatial Attention	0.173	91.58%	0.9281	0.9189	0.9145	-7.70%
Without SE Block	0.1426	94.85%	0.9543	0.95	0.9491	-4.43%
Without Residual Path	0.0893	96.29%	0.9676	0.9644	0.9643	-2.99%
Without Separable Conv	0.2948	98.95%	0.9892	0.99	0.9895	-0.33%
Baseline (Fire Module only)	0.1796	97.18%	0.9725	0.9729	0.9722	-2.10%

Bold values denote major findings in the work.

The most significant performance degradation occurred when Channel Shuffle was removed, resulting in a 10.85% drop in accuracy. This confirms that shuffling is vital for enabling information flow between channel groups after the squeeze operation, ensuring the model captures diverse feature relationships. The removal of Spatial Attention and SE Block led to accuracy drops of 7.70% and 4.43% respectively. This demonstrates that the SE block helps the model identify what features are important (channel-wise attention) and the Spatial Attention block helps to identify where the disease spots are located on the leaf (spatial-wise attention). Omitting the Residual Path (identity mapping) decreased accuracy by 2.99% and justifies its inclusion to maintain gradient stability and feature preservation during deep layer processing. Replacing the 3x3 Depthwise Separable Convolutions with standard convolutions increased the parameters from 0.17M to 0.29M (70% increase) while offering no significant accuracy gain. This validates the use of separable convolutions as a core strategy for achieving a lightweight architecture without compromising performance. Full Model represents the optimal balance between parametric efficiency and diagnostic precision.

### 7.5 Statistical analysis

#### 7.5.1 Parametric tests

##### 7.5.1.1 One-way ANOVA

One-way ANOVA is a parametric test used to examine whether there are statistically significant differences in results. **Table 9** summarizes the results of the one-way ANOVA conducted to evaluate whether statistically significant differences exist among ALNet and the pretrained models across all performance metrics. For Accuracy, Precision, Recall, F1-score and AUC, the analysis reveals high F-statistic values (ranging from 73.25 to 309.37) with corresponding p-values below 0.05, leading to the rejection of the null hypothesis for all performance metrics. This indicates that the between-group variance is substantially larger than the within-group variance, confirming that model choice has a significant impact on performance across all evaluated metrics. Overall, these findings provide robust statistical evidence that the evaluated architectures do not perform equivalently, thereby justifying the use of subsequent *post-hoc* tests to identify specific pairwise differences and to further substantiate the comparative advantage of ALNet.

**Table 9:** One-way ANOVA test results for all metrics.

Metric	Source	Sum of squares	df	Mean square	F-statistic	P-value	Significant?
Accuracy	Between Groups	0.350829	5	0.070166	73.2460	< 0.05	**Yes**
	Within Groups	0.022991	24	0.000958	–	–	
Precision	Between Groups	0.295276	5	0.059055	119.2381	< 0.05	**Yes**
	Within Groups	0.011886	24	0.000495	–	–	
Recall	Between Groups	0.352125	5	0.070425	76.7624	< 0.05	**Yes**
	Within Groups	0.022019	24	0.000917	–	–	
F1-Score	Between Groups	0.365128	5	0.073026	75.3175	< 0.05	**Yes**
	Within Groups	0.023270	24	0.000970	–	–	
AUC	Between Groups	0.060455	5	0.012091	309.3701	< 0.05	**Yes**
	Within Groups	0.000938	24	0.000039	–	–	

Bold values denote major findings in the work.

##### 7.5.1.2 Paired *t*-test

To compare analyze the Paired *t*-test results between ALNet and the pretrained models results for the accuracy are summarized in **Table 10**. The results obtained from paired *t*-test show that ALNet shows a statistically significant improvement over ResNet50 and SqueezeNet by achieving mean performance gains of 30.65% and 3.35%, respectively (*p* < 0.05). These findings support ALNet’s enhanced feature extraction capability and architectural efficiency relative to conventional convolutional frameworks. In contrast, although ALNet showed marginally higher mean performance compared to EfficientNetB0 (+4.88%), ShuffleNet (+1.57%), and SENet (+0.20%), the differences were not statistically significant (*p* > 0.05), indicating that their performances are comparable within experimental variability. Overall, the statistical analysis substantiates that ALNet achieves consistent and reliable performance enhancements over various architectures and maintains competitive results with pretrained models.

**Table 10:** Paired *t*-test results between ALNet and pretrained models for accuracy.

Comparison	Mean diff	Std dev	T-statistic	df	P-value	Significant?
**ALNet vs ResNet50**	**30.6520%**	**1.1625%**	**58.9599**	**4**	**< 0.05**	**Yes**
ALNet vs EfficientNetB0	4.8760%	6.5849%	1.6558	4	> 0.05	No
ALNet vs ShuffleNet	1.5740%	4.2013%	0.8377	4	> 0.05	No
**ALNet vs SqueezeNet**	**3.3460%**	**2.1762%**	**3.4380**	**4**	**< 0.05**	**Yes**
ALNet vs SENet	0.2000%	1.4982%	0.2985	4	> 0.05	No

Bold values denote major findings in the work.

#### 7.5.2 Non-parametric tests

##### 7.5.2.1 Friedman test

Friedman test is a non-parametric test which is used to assess whether statistically significant differences exist in the results obtained. **Table 11** shows the results of the Friedman test conducted to statistically compare ALNet with other pretrained models across multiple performance metrics. For all evaluated metrics (Accuracy, Precision, Recall, F1-score and AUC), the Friedman χ² values range from 14.71 to 17.91 with 5 degrees of freedom and the corresponding p-values are below 0.05, indicating statistical significance. These results demonstrate that there are significant differences in performance among the compared models for every metric considered. In particular, the rejection of the null hypothesis confirms that the observed variations are not due to random chance but are because of the genuine differences in model behavior. This statistical evidence supports the claim that ALNet exhibits performance characteristics that are distinct from those of the other pretrained architectures.

**Table 11:** Friedman test results for all metrics.

Metric	Friedman χ²	df	P-value	Significant?
Accuracy	14.9429	5	< 0.05	**Yes**
Precision	14.9429	5	< 0.05	**Yes**
Recall	14.7143	5	< 0.05	**Yes**
F1	14.9429	5	< 0.05	**Yes**
AUC	17.9143	5	< 0.05	**Yes**

Bold values denote major findings in the work.

##### 7.5.2.2 Wilcoxon signed-rank test

To further investigate the pairwise performance differences identified by the Friedman test, the Wilcoxon signed-rank test was employed as a non-parametric *post-hoc* analysis to compare the accuracy of ALNet against each pretrained model across the same experimental folds. **Table 12** presents the results of the Wilcoxon signed-rank test comparing the accuracy of ALNet against various pretrained models. A statistically significant improvement is observed for ALNet over ResNet50 and SqueezeNet, with mean accuracy differences of 30.65% and 3.35%, respectively, accompanied by p-values< 0.05 and exclusively positive ranks, indicating consistent performance gains across all folds. In contrast, although ALNet shows higher mean accuracy than EfficientNetB0, ShuffleNet, and SENet, the corresponding p-values exceed 0.05, suggesting that these improvements are not statistically significant and may be attributable to variability across folds. Overall, these results indicate that ALNet’s performance gains over stronger pretrained models are comparatively modest and statistically comparable, reinforcing the robustness of ALNet.

**Table 12:** Wilcoxon signed-rank test results between ALNet and pretrained models for accuracy.

Comparison	Mean diff	W statistic	Positive ranks	Negative ranks	P-value	Significant?
**ALNet vs ResNet50**	**30.6520%**	**0.0**	**15.0**	**0.0**	**< 0.05**	**Yes**
ALNet vs EfficientNetB0	4.8760%	2.0	13.0	2.0	> 0.05	No
ALNet vs ShuffleNet	1.5740%	7.0	8.0	7.0	> 0.05	No
**ALNet vs SqueezeNet**	**3.3460%**	**0.0**	**15.0**	**0.0**	**< 0.05**	**Yes**
ALNet vs SENet	0.2000%	4.0	6.0	4.0	> 0.05	No

Bold values denote major findings in the work.

### 7.6 SOTA analysis

**Table 13** shows the comparison of ALNet with SOTA methods. As seen in **Table 13**, ALNet outperforms all the other SOTA methods in all the three datasets and achieves the highest accuracy. This proves that ALNet is a highly accurate model which can be used for any plant leaf disease dataset.

**Table 13:** Comparison of the proposed model with the other state-of-the-art methods.

Study	Model/technique used	Dataset	Accuracy
Proposed methodology	ALNet	Grapevine, Apple and Cherry - PlantVillage	Grapevine - 99.78%, Apple - 99.95%, Cherry - 100%
(Kaur and Devendran, 2024)	Ensemble classifiers with hybrid feature extraction	Grapevine - PlantVillage	95.69%
(Ji et al., 2020)	UnitedModel (GoogLeNet + ResNet50)	Grapevine - PlantVillage	98.57%
(Talaat et al., 2025)	Optimized CNN (PDDA)	Grapevine - PlantVillage	99.7%
(Shantkumari and Uma, 2023)	CNN + Improved KNN	Grapevine - PlantVillage	CNN – 96.60%, KNN – 98.07%
(Zhang et al., 2025)	DLVTNet	Grapevine - PlantVillage	98.48%
(Tang et al., 2020)	ShuffleNet + SE blocks	Grapevine - PlantVillage	99.15%
(Bansal et al., 2021)	Ensemble (DenseNet121 + EfficientNetB7 + NoisyStudent)	Apple - Plant Pathology 2020 FGVC7	96.25%
(Fu et al., 2022)	Custom Lightweight CNN (Modified AlexNet + Dilated Conv + Multi-Scale + SE Attention + Global Pooling)	Apple - AI Studio dataset	97.36%
(Vishnoi et al., 2023)	Custom Lightweight CNN (Conv-3 DCNN)	Apple - PlantVillage	98%
(Jiang et al., 2025)	Optimized Deep CNN	Cherry - PlantVillage	99.2%
(Sharma and Minhas, 2024)	CNN for feature extraction + Gradient Boosting	Cherry - PlantVillage	98.9%
(Asghar et al., 2025)	HPDC-net	Potato and Tomato - PlantVillage	99.36%
(Rakib et al., 2024)	Lightweight Quantized CNN Model	PlantVillage	98%
(Khanna et al., 2024)	PlaNet	PlantVillage and two other datasets	98.51% 97.51% 98.72%
(Nnamdi and Abolghasemi, 2025)	Optimized MobileNet	PlantVillage	99%
(Akuthota et al., 2024)	Lightweight Low-Power Model made up of SqueezeNet and EfficientDet-Lite0	PlantVillage, google images and real time images	97.89%

## 8 Conclusion and future works

### 8.1 Conclusion

The proposed deep learning model, ALNet, has shown impressive classification accuracy even though the number of parameters both trainable and non-trainable is less, especially for plant leaf disease images. By incorporating novel architectural blocks from various pretrained models such as ResNet, SENet, EfficientNet, SqueezeNet and ShuffleNet.

The primary focus of the research is to classify the four classes in the grapevine leaf disease dataset into ESCA, Black Rot, Leaf Blight and Healthy. The highest achieved accuracy was about 99.78 and 100 percent on multi-class and binary classification. Then, the model was evaluated on the apple and cherry leaf dataset to check the robustness of the model. The model performed better than various pre-trained models. These results show that ALNet is effective in classifying the diseases in various plant leaf disease dataset and hence making it an impactful model revolutionizing the automation of disease prediction in the agriculture field. The model has about 0.17 million parameters and 113 layers which yields high accuracy with low computation complexity and low memory usage.

Our research has shown significant potential of lightweight CNN in achieving a high accuracy with lesser memory usage making it easy to mount on edge devices and on cloud platforms. However, one thing to note is that further research should be carried out on classifying the various leaf diseases in real life. In other words, research should be carried out to make the model to effectively classify even if the images don’t have much contrast between the background and the leaf. This research will make it easy to deploy the model in real time for farmers to use. ALNet is a successful CNN used for image classification by achieving promising results on three agriculture datasets. These findings also suggest that ALNet has the potential to solve other image classification problems as well with appropriate fine tuning.

### 8.2 Limitation and future works

Despite the ALNet’s strong performance and high parametric efficiency, certain limitations of the present study should be acknowledged.

First, although the model has been extensively validated using five-fold cross-validation across three publicly available datasets (grapevine, apple, and cherry), all experiments were conducted under controlled or artificially introduced conditions using curated datasets derived from the PlantVillage repository. These datasets contain images captured under relatively uniform lighting and background conditions or on artificially made noisy dataset, which may not fully represent real-world agricultural environments where factors such as varying light intensity, complex backgrounds, occlusions and motion blur are common and more complex in the image captured.

Second, while computational efficiency is reported in terms of model size, number of parameters, MFLOPs and training time on a GPU-based environment, the inference latency and energy consumption of ALNet have not yet been evaluated on real edge hardware. Although the lightweight design strongly suggests suitability for edge deployment, benchmarking on physical edge devices is required to fully validate real-time performance and power efficiency.

Finally, the model has been evaluated on a limited number of crop species and disease categories. While the results indicate good generalizability across similar plant leaf datasets, further validation on a broader range of crops, diseases and real-field images is necessary to confirm scalability.

Addressing these limitations will be the scope for future research. Ongoing discussions with nearby farms are being conducted to facilitate real-world data collection and deployment of the proposed system. In parallel, discussions with edge-device solution providers are underway to integrate ALNet into a deployable product, enabling systematic evaluation of inference latency, power consumption and robustness under real operational conditions. These efforts will help transition ALNet from a research prototype to a practical, field-ready product for farmers.

## Data Availability

The original contributions presented in the study are included in the article/supplementary material. Further inquiries can be directed to the corresponding author.
